# EGFRvIII Reduces Neural Stem/Progenitor Marker Expression in GFAP-Negative iNSCs: Evidence for a Context-Dependent Cellular Response

**DOI:** 10.3390/ijms27188118

**Published:** 2026-09-12

**Authors:** Aneta Włodarczyk, Cezary Tręda, Dagmara Grot, Ewelina Stoczyńska-Fidelus, Piotr Rieske

**Affiliations:** 1 Department of Tumor Biology, Medical University of Lodz, Zeligowskiego 7/9, 90-752 Lodz, Polandpiotr.rieske@umed.lodz.pl (P.R.); 2 Department of Molecular Biology, Medical University of Lodz, Zeligowskiego 7/9, 90-752 Lodz, Poland

**Keywords:** glioblastoma, EGFRvIII, induced neural stem cells, SOX2, nestin, GFAP, cell-state remodeling

## Abstract

Epidermal growth factor receptor variant III (EGFRvIII) is a constitutively active EGFR deletion variant found in a subset of glioblastomas (GB). Its association with stem-like tumor populations is well documented, but the outcome of EGFRvIII expression may vary with the developmental and molecular state of the recipient cell. Here, we used human induced pluripotent stem cell (iPSC)-derived, glial fibrillary acidic protein (GFAP)-negative-induced neural stem cells (iNSCs) to explore how this receptor variant affects a defined neural stem/progenitor background. EGFRvIII was introduced either constitutively by lentiviral transduction or through a doxycycline-responsive Tet-On system. Both experimental approaches were associated with remodeling of the neural stem/progenitor marker profile, with reduced SRY-box transcription factor 2 (SOX2) expression in the inducible model and significant reductions in both SOX2 and nestin in the constitutive model. Constitutive expression was also associated with increased senescence-associated β-galactosidase (SA-β-Gal) activity, while doxycycline induction produced a distinct change in cell-number dynamics at low doxycycline concentration. Thus, in this experimental setting, EGFRvIII was linked to substantial remodeling of the SOX2+/nestin+ marker profile. These observations do not define a differentiation pathway, prove cellular senescence or malignant transformation, or identify the cell of origin of EGFRvIII-positive GB. Rather, they support the view that the cellular response to EGFRvIII should be considered in relation to the specific neural-glioblastoma precursor context.

## 1. Introduction

Glioblastoma (GB) remains the most aggressive primary malignant brain tumor in adults, with limited survival despite combined treatment approaches [1,2,3,4]. Its biological diversity is evident not only at the genetic level but also in the range of cellular states present within individual tumors. The identity of the normal or precursor cell in which gliomagenic alterations first become effective is still debated. Neural stem and progenitor cells, oligodendrocyte precursor cells, astrocytic populations, radial glia, and mature neural cells capable of reprogramming have all been considered as candidate starting populations [5,6,7,8,9]. Rather than supporting a single universal origin, these observations are compatible with a heterogeneous-origin framework in which different neural cell states may show different susceptibility to oncogenic alterations. Accordingly, the consequences of a given glioma-associated alteration may depend partly on the developmental and molecular state of the cell in which it is acquired [8,9].

One recurrent alteration in GB is epidermal growth factor receptor variant III (EGFRvIII), a constitutively active deletion form of EGFR. EGFRvIII occurs in heterogeneous tumor cell populations and has been linked to aggressive behavior and glioblastoma stem-like features [10,11,12,13,14]. At the same time, its biological output is not uniform and appears to depend on the molecular background of the cells in which it is present [10,13,14]. EGFRvIII has been described as an alteration that can occur relatively early in GB evolution, although its abundance may vary during subsequent tumor development [14,15]. Therefore, the behavior of EGFRvIII in an established malignant population cannot automatically be extrapolated to a neural precursor that has not undergone tumor-associated evolution.

SOX2 and nestin are commonly used to describe neural stem/progenitor identity and are also present in subsets of GB, including stem-like tumor populations [10,16,17]. Neural precursor populations, however, are heterogeneous, and oncogenic perturbations may produce different outcomes depending on lineage and developmental state [5,6,7,8,9]. The iNSCs examined here were derived from human iPSCs and were predominantly SOX2- and nestin-positive, with negligible glial fibrillary acidic protein (GFAP) staining and only a small microtubule-associated protein 2 (MAP2)-positive fraction. We therefore used these cells as a defined GFAP-negative neural stem/progenitor setting in which to examine the response to EGFRvIII.

This exploratory study asked how expression of EGFRvIII changes the marker profile and selected cellular features of GFAP-negative iNSCs. To reduce dependence on a single expression strategy, we examined both a constitutive lentiviral model and a doxycycline-inducible Tet-On model. These complementary systems also allowed us to compare a sustained-expression setting with a controllable induction setting, while recognizing that the two approaches are not quantitatively equivalent. The experiments were designed to characterize the response of this particular iNSC system, rather than to resolve the cell of origin of GB or the chronological position of EGFRvIII acquisition during gliomagenesis.

## 2. Results

**Figure 1:**
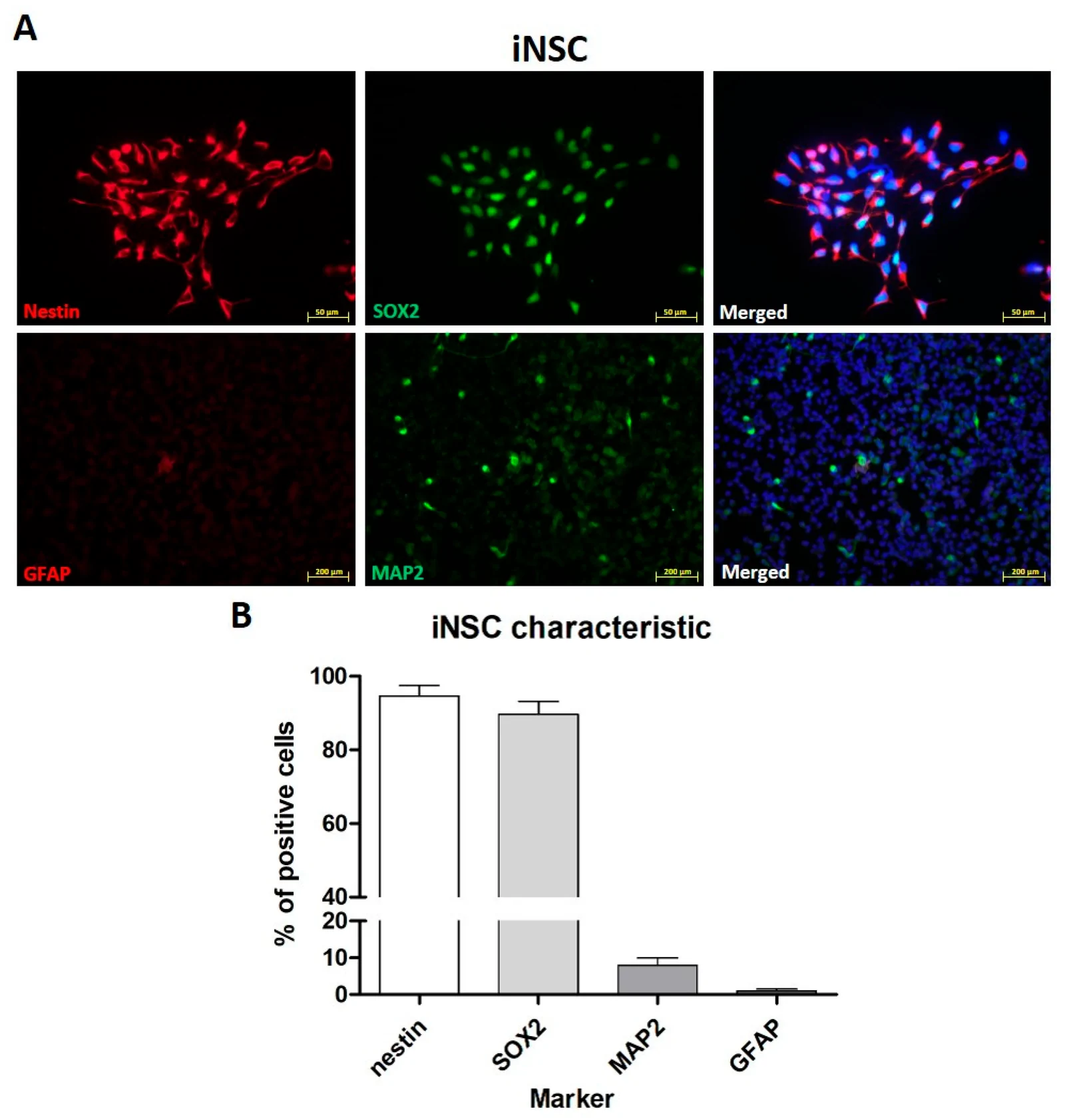
Phenotypic characterization of GFAP-negative iNSCs. (**A**) Representative immunocytochemical images showing nestin, SOX2, MAP2, and GFAP expression. Images were acquired at 40× magnification for nestin and SOX2 and 20× for MAP2 and GFAP. Exposure times were DAPI, 10 ms; SOX2, 300 ms; nestin, 300 ms; MAP2, 400 ms; and GFAP, 300 ms. (**B**) Quantitative characterization of marker-positive cells. The majority of cells were nestin- and SOX2-positive, a small population was MAP2-positive, and GFAP expression was negligible. Data were obtained from three independent biological experiments (*n* = 3), with five microscopic fields analyzed per condition in each experiment.

First, the starting iNSC population was phenotypically characterized. The majority of cells were positive for the neural stem/progenitor markers nestin and SOX2, whereas approximately 5% of cells were MAP2-positive and GFAP expression was practically undetectable (Figure 1A,B). These findings define the starting population used in this study as a predominantly SOX2+/nestin+, GFAP-negative iNSC model.

**Figure 2:**
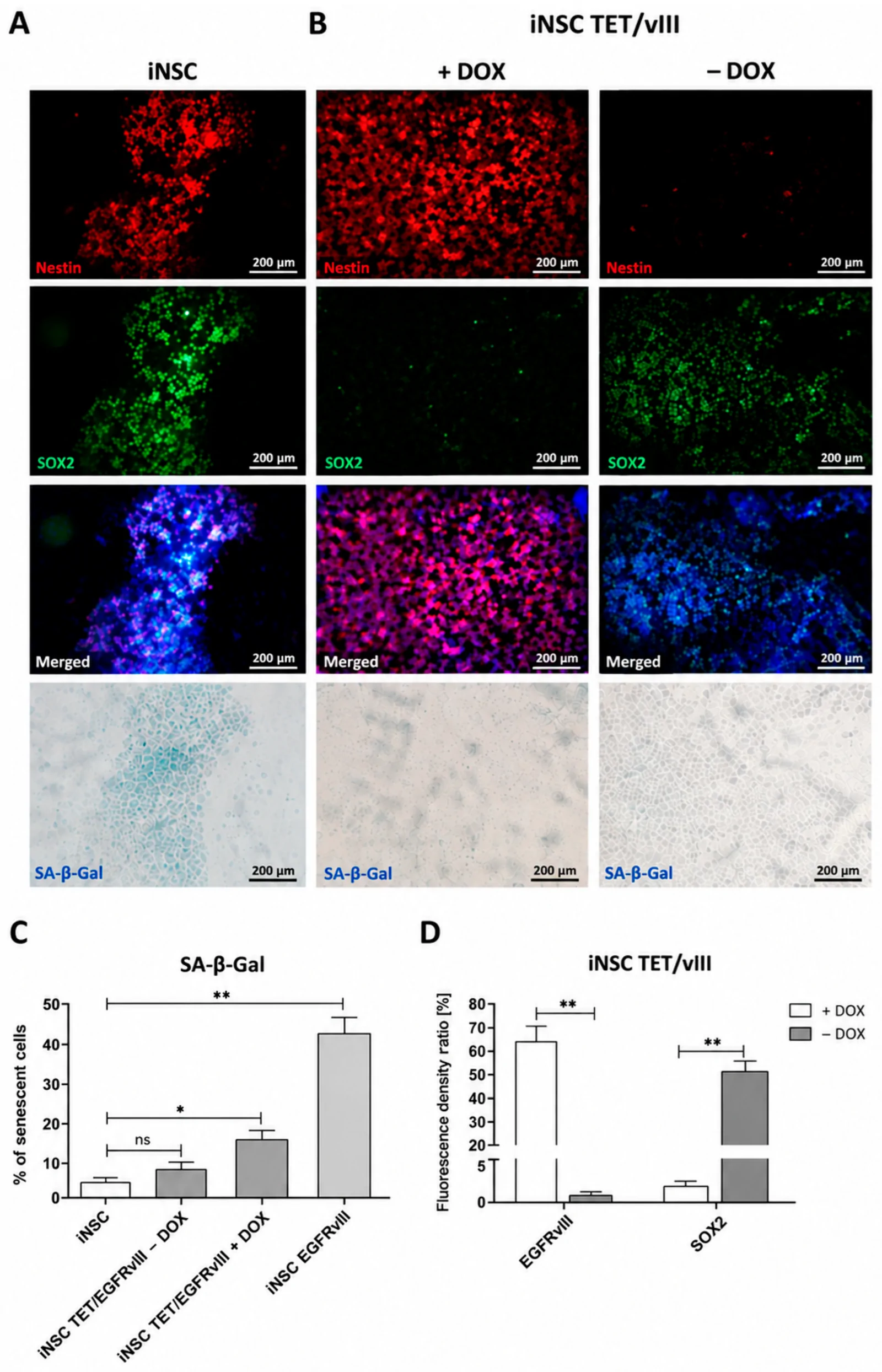
Phenotypic response of the inducible iNSC-TET/vIII model to EGFRvIII induction. (**A**,**B**) Representative immunocytochemical images of iNSCs and iNSC-TET/vIII cells maintained with or without doxycycline (1 µg/mL) for 14 days. (**C**) Quantification of SA-β-Gal-positive cells. (**D**) Quantitative fluorescence analysis of EGFRvIII and SOX2 in iNSC-TET/vIII cells with and without doxycycline. EGFRvIII signal increased following induction (*p* = 0.0037), whereas SOX2 signal decreased (*p* = 0.0038; two-tailed paired Student’s *t*-tests; *n* = 3 independent biological experiments). Five microscopic fields were analyzed per condition in each experiment. In all analyses, the statistical significance of results was determined as follow: *p* > 0.05 (ns), *p* ≤ 0.05 (*) and *p* ≤ 0.01 (**).

We next examined the doxycycline-inducible iNSC-TET/vIII model. After 14 days of treatment with 1 µg/mL doxycycline, EGFRvIII immunoreactivity increased markedly, while SOX2 expression was reduced relative to the matched non-induced condition (Figure 2A–D). Quantitative fluorescence analysis confirmed an increase in EGFRvIII signal (*p* = 0.0037) and a reduction in SOX2 signal (*p* = 0.0038). SA-β-Gal-positive cells were also more frequent after induction than in the non-induced control (Figure 2A–C). Because SA-β-Gal positivity alone is not sufficient to establish definitive senescence, this observation is interpreted as a senescence-associated phenotype rather than conclusive cellular senescence.

**Figure 3:**
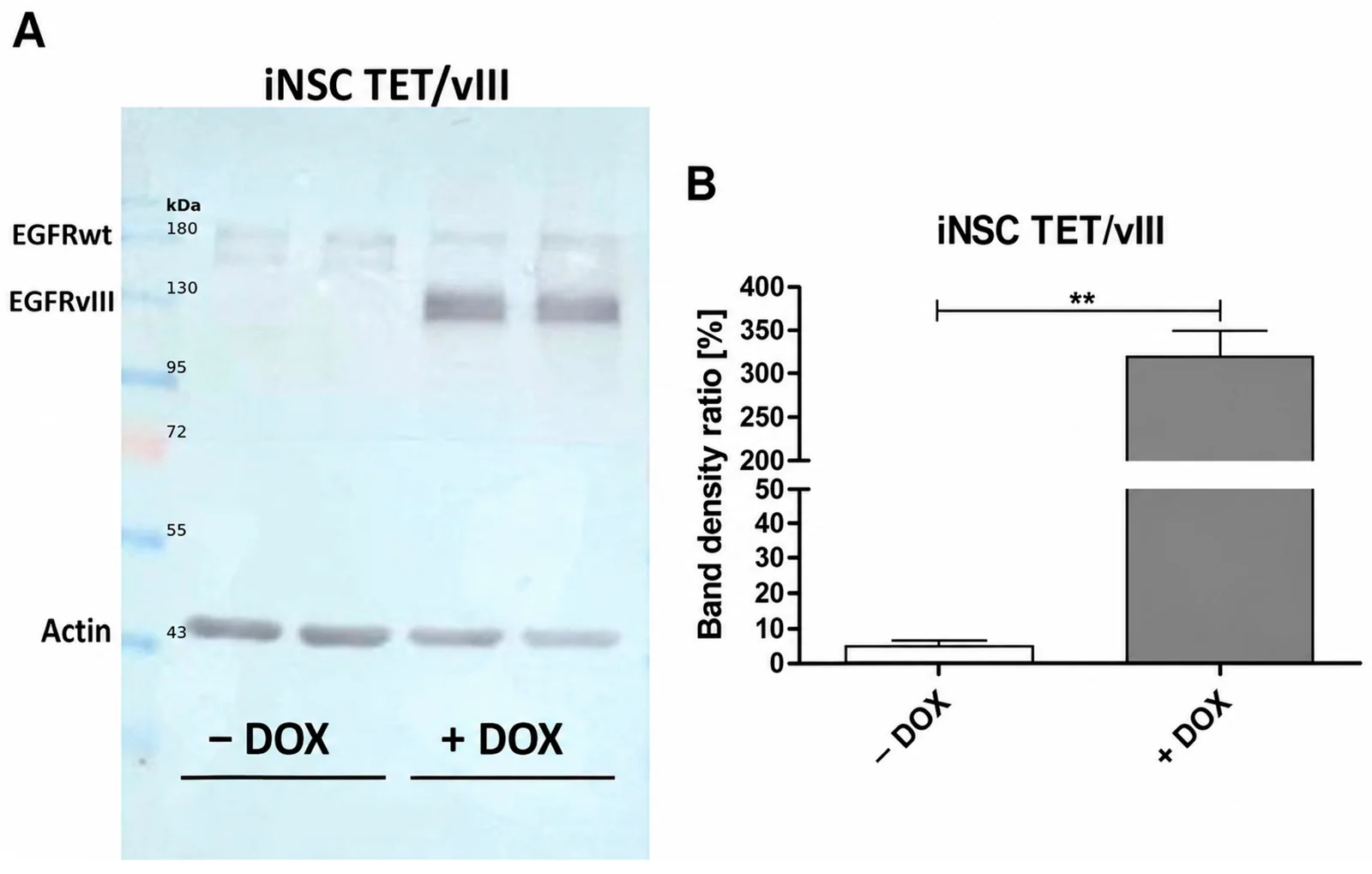
Confirmation of EGFRvIII expression in GFAP-negative iNSC-TET/vIII cells. (**A**) Representative Western blot after 14 days of culture with or without doxycycline (1 µg/mL). β-actin was used as the loading control, and molecular weights were estimated using the Color Protein Standard, Broad Range (P7719S, New England Biolabs, Ipswich, MA, USA). The EGFRvIII signal appears as a closely migrating doublet; both immunoreactive bands were included in densitometric quantification. (**B**) EGFRvIII densitometry normalized to β-actin from three independent biological experiments (*n* = 3; mean ± SD). In all analyses, the statistical significance of results was determined as follow: *p* ≤ 0.01 (**).

Induction of EGFRvIII in the iNSC-TET/vIII model was independently confirmed by Western blotting after 14 days of doxycycline treatment (Figure 3A). The blot shown is representative of three independent biological experiments, and densitometric analysis included all three replicates (Figure 3B). The EGFRvIII immunoreactive signal appeared as a closely migrating doublet. The present experiments do not establish the molecular basis of the doublet; it may reflect differences in receptor processing or post-translational modification.

**Figure 4:**
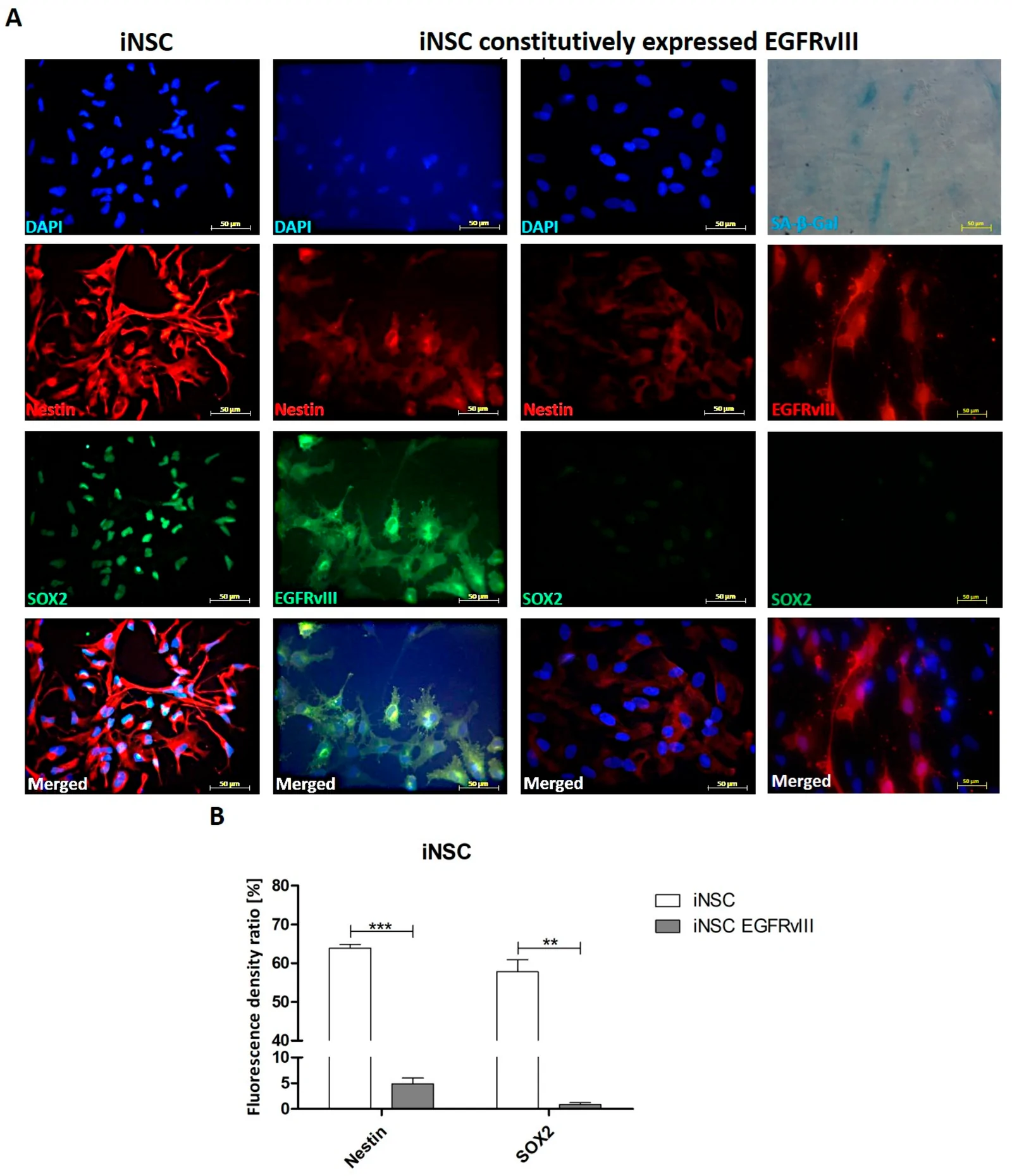
Effect of constitutive EGFRvIII expression on neural stem/progenitor markers in GFAP-negative iNSCs. (**A**) Representative images of control iNSCs and iNSCs constitutively expressing EGFRvIII, including nestin and SOX2 immunocytochemistry and SA-β-Gal staining. (**B**) Quantitative analysis of nestin and SOX2 expression. Constitutive EGFRvIII expression was associated with a significant reduction in nestin (*p* = 0.00034) and SOX2 (*p* = 0.00316) compared with matched control iNSCs (two-tailed paired Student’s *t*-tests). Data were obtained from three independent biological experiments *(n* = 3), with five microscopic fields analyzed per condition in each experiment. In all analyses, the statistical significance of results was determined as follow: *p* ≤ 0.01 (**) and *p* ≤ 0.001 (***).

In the constitutive iNSC/EGFRvIII model, stabilization was frequently difficult, and the surviving population showed marked remodeling of the starting neural stem/progenitor marker profile. Quantitative immunocytochemistry demonstrated a pronounced reduction in nestin expression relative to control iNSCs (*p* = 0.00034) and a corresponding reduction in SOX2 expression (*p* = 0.00316; Figure 4A,B). Increased SA-β-Gal activity was also observed in the constitutive model. These findings are therefore described as reduced neural stem/progenitor marker expression accompanied by a senescence-associated phenotype, without assigning a specific differentiation trajectory or definitive senescent state.

**Figure 5:**
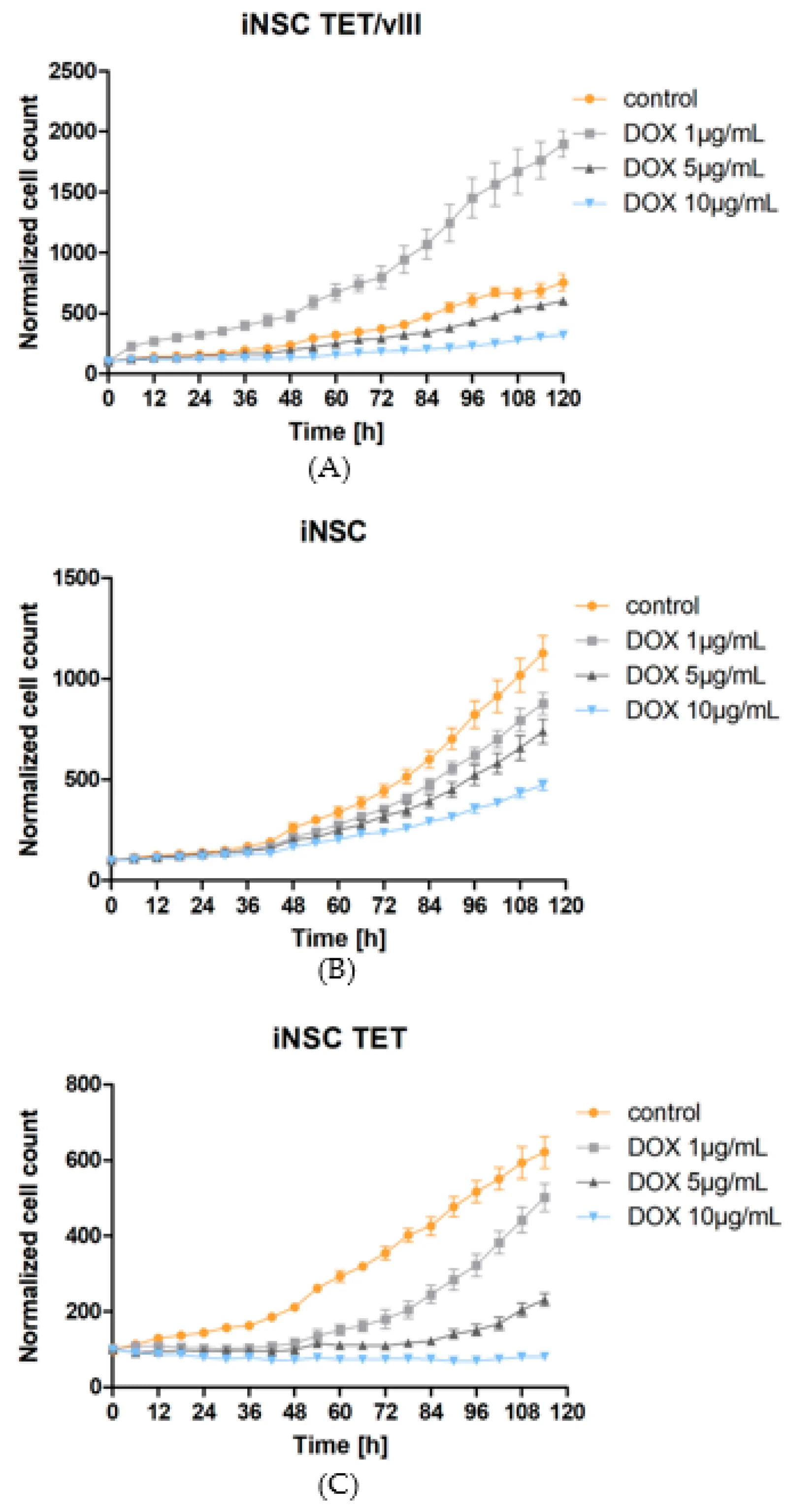
Effect of doxycycline concentration on cell-number dynamics in GFAP-negative iNSC models. Cell numbers were monitored by real-time imaging and normalized to the corresponding baseline value at 0 h, which was set to 100% for each independent experiment. Data represent five independent biological experiments (*n* = 5). (**A**) iNSC-TET/vIII cells showed an increased cell-number trajectory at 1 µg/mL doxycycline relative to the non-induced condition (treatment effect, *p* = 0.0068; time × treatment interaction, *p* < 0.0001). (**B**) Parental iNSCs and (**C**) iNSC-TET control cells demonstrated that doxycycline itself influences cell-number dynamics. Direct comparison of the normalized 0–114 h AUC demonstrated greater cell-number expansion in iNSC-TET/vIII + 1 µg/mL doxycycline than in iNSC-TET + 1 µg/mL doxycycline (*p* = 0.0013). Reduced cell-number expansion at higher doxycycline concentrations is reported descriptively and is not interpreted as a direct measure of cytotoxicity.

Real-time imaging was used to compare cell-number dynamics in the inducible model and the corresponding controls (Figure 5). In iNSC-TET/vIII cells, 1 µg/mL doxycycline significantly altered the normalized cell-number trajectory relative to the non-induced condition (repeated-measures two-way ANOVA: treatment effect, F(1,4) = 26.35, *p* = 0.0068; time × treatment interaction, F(19,76) = 10.19, *p* < 0.0001) (Figure 5A). In parental iNSCs, the main treatment effect at 1 µg/mL was not significant (*p* = 0.2504), although the time × treatment interaction was significant (*p* = 0.0007) (Figure 5B). In iNSC-TET control cells lacking EGFRvIII, doxycycline produced a different response, with a treatment effect in the opposite direction (F(1,4) = 9.24, *p* = 0.0384; time × treatment interaction, *p* < 0.0001) (Figure 5C). Consistently, direct comparison of the normalized 0–114 h AUC showed greater cell-number expansion in doxycycline-treated iNSC-TET/vIII cells than in doxycycline-treated iNSC-TET controls (Welch’s *t*-test, *p* = 0.0013). At higher doxycycline concentrations, cell-number expansion was reduced. Because doxycycline itself influenced the control populations, these findings are interpreted as an EGFRvIII-associated differential cell-number response rather than as an effect attributable exclusively to EGFRvIII.

## 3. Discussion

Our data show that the response to EGFRvIII in the GFAP-negative iNSC model is accompanied by a substantial change in neural stem/progenitor markers. Both experimental systems showed remodeling of the neural stem/progenitor marker profile. In the inducible model, quantitative analysis demonstrated reduced SOX2 expression following EGFRvIII induction, whereas the constitutive model showed significant reductions in both SOX2 and nestin. This result does not rule out GFAP-negative neural stem/progenitor cells as a possible source population for EGFRvIII-positive GB. Instead, it indicates that introducing EGFRvIII into the particular precursor state studied here destabilizes its initial neural stem/progenitor marker profile.

This response differs from the association between EGFRvIII and stem-like properties described in established GB. For example, Emlet et al. identified EGFRvIII-positive human GB cells enriched for stem/progenitor features and reported strong self-renewal and tumor-initiating capacity in an EGFRvIII-positive stem-like fraction [10]. Loss of EGFR/EGFRvIII during differentiation of glioblastoma stem-like cells has likewise been linked to reduced stem-like and tumorigenic potential [18]. These studies address receptor behavior after malignant transformation, whereas our experiments introduce EGFRvIII into an iPSC-derived neural precursor background. The different outcomes can therefore be viewed as evidence of different cellular contexts rather than mutually exclusive observations. In particular, an association between EGFRvIII and stemness in an established tumor does not demonstrate that acquisition of EGFRvIII alone will preserve the same state in a non-tumor neural precursor.

The marker changes observed here also do not reveal what state the cells subsequently acquire. A decrease in SOX2 and nestin can accompany several processes, including differentiation, cellular stress, preferential survival of a subpopulation, or another form of state transition. This uncertainty is especially relevant to GB, where multiple neural-progenitor-like, oligodendrocyte-progenitor-like, astrocyte-like, and mesenchymal-like malignant cell states can coexist and show substantial plasticity [19]. In the present experiments, MAP2 and GFAP were used to define the starting iNSC population; they were not used to assign a post-EGFRvIII fate. We therefore interpret the data as evidence of remodeling of the initial neural stem/progenitor profile, without naming the resulting cellular identity.

This context dependence is particularly relevant in a disease characterized by extensive cellular plasticity. The coexistence of several malignant cell states within individual glioblastomas means that a change in a limited marker set should not be interpreted as movement along a single predetermined differentiation pathway [19]. Instead, the present findings can be viewed as an example of how the same oncogenic alteration may be accommodated differently according to the state of the recipient cell and the molecular alterations already present. This interpretation is consistent with the broader view that EGFRvIII-associated phenotypes in established tumor cells and responses to newly introduced EGFRvIII in neural precursors need not be identical.

The study has several constraints. Only one iPSC-derived cellular background was examined. Independent biological repeats demonstrate reproducibility within that background but cannot capture variability between donors or independently derived iNSC lines. The starting population was defined operationally by strong SOX2/nestin expression, a small MAP2-positive fraction and negligible GFAP staining; future work would benefit from a wider neural precursor marker panel and additional iNSC backgrounds. In addition, SA-β-Gal was used as an indicator associated with senescence rather than as proof of a complete senescence program. Current recommendations emphasize that no single marker is sufficient to establish cellular senescence and support combining cell-cycle arrest measures with additional senescence-associated readouts [20]. Confirmation in this model would therefore require complementary measurements such as p16INK4a, p21CIP1, proliferative capacity, SASP components, γH2AX or Lamin B1. Finally, the live-imaging assay measures net population size and cannot distinguish slower division from cell loss, so it does not provide evidence of apoptosis.

Expression magnitude represents another limitation. We did not quantitatively align EGFRvIII abundance between the constitutive and inducible systems or benchmark either model against endogenous receptor levels in EGFRvIII-positive human GB. Transduction efficiency was also not formally quantified in the constitutive model. Because this model used an MOI of 100, receptor abundance may have exceeded physiological levels, and the strong marker reduction and elevated SA-β-Gal activity could partly reflect expression magnitude or prolonged exposure. A vector-only population transduced at the same MOI was not available for the constitutive experiments, so effects related to viral load or transduction stress cannot be excluded. Stabilization-related selection and differences in expression duration may further contribute to the distinct behavior of the two models. At the same time, the divergence between sustained and inducible expression is biologically noteworthy: it raises the hypothesis that not only the presence of EGFRvIII, but also the magnitude, duration, and temporal dynamics of its expression may influence the cellular response. The present experiments do not resolve these variables, and direct measurement of EGFRvIII abundance over time will be required to test this possibility.

The signaling events linking EGFRvIII to the observed marker changes were not resolved in this study. Canonical EGFR pathway activation is not necessarily a uniform readout of EGFRvIII across cellular settings. In earlier work with DK-MG glioblastoma cells, populations with different endogenous EGFRvIII levels did not show matching differences in PI3K/AKT activation [18]. In a later study, increasing EGFRvIII experimentally failed to reproduce the phenotype associated with high endogenous receptor expression, whereas K-RASG12V generated a distinct phenotype together with ERK and AKT activation [13]. These findings are compatible with context-dependent, potentially non-canonical EGFRvIII signaling. However, since signaling was not measured directly in the current iNSCs, no specific pathway can be assigned to the SOX2/nestin reduction. A broader mechanistic analysis will be required.

The inducible model introduces additional experimental considerations. Doxycycline changed cell-number trajectories in control cells, and receptor abundance was not quantified at each doxycycline concentration. The concentration series therefore describes a cellular response to the inducible system and should not be read as a calibrated EGFRvIII protein dose–response. Moreover, the cultures were not maintained under specifically tetracycline-free conditions, so a small degree of basal Tet-On activity cannot be excluded. Such factors are recognized limitations of inducible expression approaches and should be considered when comparing the Tet-On and constitutive models [21,22].

Overall, EGFRvIII expression in this GFAP-negative iNSC model was associated with erosion of the initial SOX2+/nestin+ neural stem/progenitor marker pattern and, in the constitutive system, greater SA-β-Gal activity. The experiments demonstrate phenotypic remodeling but do not define the final cell state, establish tumorigenicity, identify a GB cell of origin, or determine when EGFRvIII is acquired during gliomagenesis. The differences between the experimental systems instead point to cellular background, expression level and exposure duration as variables that may shape the response to EGFRvIII. Future work should therefore compare independently derived neural precursor backgrounds, more physiological receptor levels and relevant cooperating GB alterations, while directly examining differentiation, stress, apoptosis, senescence, and signaling readouts. Such studies would help determine which aspects of the response observed here are specific to this model and which reflect broader principles of EGFRvIII biology.

## 4. Materials and Methods

### 4.1. Generation of Induced Neural Stem Cells

Human-induced pluripotent stem cells (CLTH/hIPS, cat. no. CL 05001; Celther Polska Ltd., Lodz, Poland) served as the source material. The supplier reports expression of SOX2, OCT4, NANOG, TRA-1-81 and TRA-1-60 together with the capacity for differentiation toward all three germ layers. CLTH/hIPS cells were expanded in Essential 8 medium on Geltrex-coated culture dishes (Life Technologies, Carlsbad, CA, USA). Neural induction was then carried out with PSC Neural Induction Medium (Gibco, Waltham, MA, USA) following the manufacturer’s procedure. After induction, iNSCs were propagated on Geltrex in Neural Expansion Medium composed of Neurobasal Medium and Advanced DMEM/F12 (1:1) supplemented with Neural Induction Supplement (1:50; Life Technologies, Carlsbad, CA, USA). Experiments used iNSCs between passages 2 and 5. Mycoplasma testing was performed routinely with the Mycoplasma PCR Detection Kit (Symbios, Gdansk, Poland); all cultures included in this study were negative.

### 4.2. Genetic Constructs and Lentiviral Vectors

EGFRvIII cDNA was amplified with primers bearing attB1 and attB2 recombination sites: GGGGACAAGTTTGTACAAAAAAGCAGCGTATGCGACCCTCCGGGACGGCC and GGGGACCACTTTGTACAAGAAAGCTGGGTTGCTCCAATAAATTCACTGC. PCR products were resolved by agarose electrophoresis and purified with the NucleoSpin^®^ Gel and PCR Clean-up system (Macherey-Nagel, Duren, Germany). Gateway cloning (Invitrogen, Waltham, MA, USA) was used to introduce the amplicon first into pENTR through a BP reaction and subsequently into pLV1-puro-DEST downstream of the CMV promoter through an LR reaction. The final coding sequence was verified using an Applied Biosystems 3130 Genetic Analyzer (Thermo Fisher Scientific, Waltham, MA, USA).

### 4.3. Lentiviral Transduction of Induced Neural Stem Cells

For constitutive expression, GFAP-negative iNSCs were exposed to lentiviral particles carrying pLV1-puro-EGFRvIII at an MOI of 100, using the previously reported procedure [23]. This population is referred to as iNSC/EGFRvIII. Following stabilization, cells were allowed to proceed to the first post-stabilization passage, and molecular and phenotypic measurements were obtained 14 days after stabilization.

A separate inducible population (iNSC-TET/vIII) was established with a Tet-On lentiviral system according to the approach previously used for inducible neural stem cells [24]. In parallel, an iNSC-TET control containing the Tet system but an empty construct without EGFRvIII was maintained to distinguish responses associated with doxycycline/Tet exposure from those associated with EGFRvIII.

### 4.4. Induction of EGFRvIII Expression in iNSC-TET/vIII Cells

Induction in iNSC-TET/vIII cultures was initiated at the second passage after stabilization by adding doxycycline (1 µg/mL; A&A Biotechnology, Gdansk, Poland) to Neural Expansion Medium. Fresh doxycycline-containing medium was supplied every 48 h. Unless a different interval is specified for an individual experiment, cells were analyzed after 14 consecutive days of induction.

### 4.5. Immunofluorescence Analysis

For immunocytochemistry, 10,000 cells were plated per well on Geltrex-coated 4-well plates (Sigma-Aldrich, Burlington, MA, USA). The staining procedure followed our previously published protocol [25]. Primary antibodies were anti-nestin (1:500; Santa Cruz Biotechnology, Dallas, TX, USA), anti-SOX2 (1:300; Abcam, Cambridge, Great Britain), anti-EGFRvIII (L8A4, 1:300; Kerafast, Boston, MA, USA), anti-MAP2 (1:100; Santa Cruz Biotechnology, Dallas, TX, USA), and anti-GFAP (1:1000; Abcam, Cambridge, Great Britain). Alexa Fluor 488 anti-rabbit and Alexa Fluor 594 anti-mouse antibodies (Life Technologies, Carlsbad, CA, USA) were each diluted 1:500. Nuclei were counterstained during mounting with ProLong Gold Antifade Reagent containing DAPI (Molecular Probes, Waltham, MA, USA). Images were collected on an MN-800 FL fluorescence microscope (OPTA-TECH, Warsaw, Poland). ImageJ (ij153) was used for single-cell fluorescence measurements: each cell was delineated as a region of interest, local background was subtracted, and marker intensity was expressed relative to the corresponding DAPI signal. Three independent biological experiments were analyzed (*n* = 3). For every condition, five non-overlapping fields were measured in each experiment and averaged before inferential statistics were applied.

### 4.6. Senescence-Associated β-Galactosidase (SA-β-Gal) Assay

SA-β-Gal activity was assessed with the Senescence Cells Histochemical Staining Kit (Sigma-Aldrich, Burlington, MA, USA). Cultures were rinsed with PBS, fixed for 10 min in cold 4% PFA, rinsed again, and then exposed to freshly prepared staining solution for 12 h at 37 °C in the absence of CO_2_. After a final PBS wash, images were recorded with an Olympus CKX41 light microscope. Because this assay alone does not establish a complete senescence program, SA-β-Gal positivity was treated only as a senescence-associated readout.

### 4.7. Western Blot Analysis

Protein analysis followed the Western blot procedure reported previously [23]. Primary antibodies against EGFR (A10, 1:500; Santa Cruz Biotechnology, Dallas, TX, USA) and β-actin (MAB1501, 1:4000; Millipore, Burlington, MA, USA) were used together with anti-rabbit (sc-2004, 1:4000) and anti-mouse (sc-2005, 1:4000) secondary antibodies from Santa Cruz Biotechnology, Dallas, TX, USA. Signal development used the Opti-4CN Substrate Kit (Bio-Rad, Hercules, CA, USA). The Color Protein Standard, Broad Range (P7719S; New England Biolabs, Ipswich, MA, USA) provided molecular-weight reference points. Three independent biological experiments were performed. Densitometric measurements were obtained in ImageJ, with EGFRvIII intensity expressed relative to β-actin in the same sample. The EGFRvIII immunoreactivity resolved as two closely migrating bands; both were included when calculating the EGFRvIII signal.

### 4.8. Real-Time Observation of Cells

For longitudinal imaging, GFAP-negative iNSCs, iNSC-TET controls, and iNSC-TET/vIII cells were plated at 40,000 cells per well in Geltrex-coated 6-well plates and placed in a Nikon BioStation CT. Bright-field images were collected at 6 h intervals for as long as 120 h. Cell counts were used to describe changes in population size over time. Five independent biological experiments were performed (*n* = 5). Within each experiment and condition, every time point was divided by the corresponding count at 0 h, which was defined as 100%. Because this readout reflects net cell-number change, it cannot by itself separate altered division from cell loss and was not interpreted as an apoptosis assay.

### 4.9. Statistical Analysis

All statistical calculations were repeated during revision from the original raw measurements using SciPy 1.17.0 and statsmodels 0.14.6; ImageJ was used for fluorescence and densitometric measurements. For immunocytochemistry, the five fields collected in each experiment were first averaged, leaving three independent biological values (*n* = 3) for each condition. Matched two-condition comparisons were evaluated with two-sided paired Student’s *t*-tests, and exact *p* values are provided for the principal comparisons. The longitudinal cell-number dataset comprised five independent experiments (*n* = 5) and was assessed with repeated-measures two-way ANOVA, treating time and doxycycline exposure as repeated factors. To compare doxycycline-treated iNSC-TET/vIII and iNSC-TET cells over their shared 0–114 h interval, an area under the normalized curve (AUC) was calculated separately for each experiment and the resulting AUC values were compared with a two-sided Welch’s *t*-test. Unless indicated otherwise, values are reported as mean ± SD. Given the limited number of biological replicates in image-based analyses, formal normality tests were not used to claim distributional normality, and inferential results were interpreted conservatively.

## Data Availability

The original contributions presented in this study are included in the article. Further inquiries can be directed to the corresponding author.

## Abbreviations

The following abbreviations are used in this manuscript:

EGFR	epidermal growth factor receptor
EGFRvIII	epidermal growth factor receptor variant III
GB	glioblastoma
GFAP	glial fibrillary acidic protein
iPSC	induced pluripotent stem cell
iNSC	induced neural stem cell
MAP	microtubule-associated protein 2
NSC	neural stem cell
SA-β-Gal	senescence-associated β-galactosidase
SOX2	SRY-box transcription factor 2
